# Prévalence et facteurs de risque des pseudarthroses traumatiques des os longs des membres à l’Hôpital Matanda en ville de Butembo à l’Est de la République Démocratique du Congo

**DOI:** 10.11604/pamj.2021.40.192.28799

**Published:** 2021-11-30

**Authors:** Ernest Badako Mogonza, Aimé Lukwamirwe Vahamwiti, Amos Sivulyamwenge Kaghoma, Franck Katembo Sikakulya, Emmanuel Kabuyahia Kamenge, Sévérin Uwonda Akinja

**Affiliations:** 1 Faculté de Médecine, Université Catholique du Graben, Butembo, République Démocratique du Congo,; 2 Département de Chirurgie, Hôpital Matanda, Butembo, République Démocratique du Congo,; 3 Département de Chirurgie, Cliniques Universitaires du Graben, Butembo, République Démocratique du Congo,; 4 Faculty of clinical Medicine and dentistry, Department of Surgery, Kampala International University Western Campus, Ishaka-Bushenyi, Uganda,; 5 Faculté de Médecine, Université Officielle de Mbuji-Mayi, Mbuji-Mayi, République Démocratique du Congo

**Keywords:** Pseudarthrose traumatique, os longs, membres, hôpital Matanda, Butembo, Traumatic pseudarthrosis, long bones, limbs, Matanda Hospital, Butembo

## Abstract

La pseudarthrose est très redoutée et très difficile à traiter, même par le chirurgien orthopédiste. Le mieux à faire est toujours de la prévenir. L´objectif de notre étude était de déterminer la prévalence et les facteurs de risque des pseudarthroses traumatiques des os longs des membres en ville de Butembo. L´étude a été rétrospective sur un échantillon de 36 patients avec pseudarthrose traumatique, tirés d´une population de 968 patients avec fracture d´un os long de membre à l´hôpital Matanda, en ville de Butembo, à l´Est de la République Démocratique du Congo, du 01/06/2016 au 31/05/2019. Les données ont été tirées des dossiers d´hospitalisation. Nous avons calculé la fréquence et utilisé un modèle de régression logistique unifactorielle et multifactorielle pour explorer les facteurs associés à la pseudarthrose. La fréquence des pseudarthroses était de 3,72%. Le type de pseudarthrose le plus fréquent était le type flottant (69,44%). Les facteurs de risque retenus étaient: les professions pousse-pousseur (ORa: 4,60; IC à 95% 1,04-15,21; p = 0,023) et cultivateur (ORa: 2,31; IC à 95% 1,17-4,68; p = 0,008), la malnutrition (ORa: 5,83; IC à 95% 1,87-15,62; p = 0,004), l´intoxication au tabac (ORa: 6,70; C à 95% 1,84-20,11; p = 0,003) et à alcool + tabac (ORa: 4,74; IC à 95% 2,17-9,89; p < 0,001), le traumatisme par balle (ORa: 6,70; IC à 95% 1,84-20,11; p = 0,003), la fracture ouverte (ORa: 4,35; IC à 95% 2,17-9,12; p < 0,001), l´infection au foyer de fracture (ORa: 3,10; IC à 95% 1,03-7,95; p = 0,023); le soignant tradipraticien (ORa: 12,18; IC à 95% 5,74-25,37; p < 0,001), le médecin généraliste (ORa: 8,33; IC à 95% 1,77-30,31; p = 0,006) et l´absence de radiographie initiale (ORa: 12,21; IC à 95% 5,92-24,96; p < 0,001). Les pseudarthroses des os longs des membres sont fréquentes à Butembo. La connaissance et l´éviction de leurs facteurs de risque pourraient être une voie de solution efficace.

## Abstract (en)

Pseudarthrosis is a dreadful condition because it is very difficult to treat even for the orthopedic surgeons. The best thing to do is always to prevent it. The purpose of our study was to determine the prevalence and risk factors for traumatic long-bone pseudarthrosis in the city of Butembo. We conducted a retrospective study of 36 patients with traumatic pseudarthrosis. Data were collected from the hospital records of 968 patients with long-bone fractures at the Matanda Hospital, in the town of Butembo, eastern Democratic Republic of the Congo from 01/06/2016 to 31/05/2019. We calculated the rate of pseudarthrosis and used a single- and multiple logistic regression model to investigate factors associated with pseudarthrosis. The rate of pseudarthrosis was 3.72%. The most common type of pseudarthrosis included floating injuries (69.44%). Risk factors were: working as a rickshaw driver (aOR: 4.60; aCI 95% 1.04-15.21; p = 0.023) and a cultivator (aOR: 2.31; aCI 95% 1.17-4.68; p = 0.008), malnutrition (aOR: 5.83; aCI 95% 1.87-15.62; p = 0.004), tobacco poisoning (aOR: 6.70; aCI 95% 1.84-20.11; p = 0.003) and tobacco+ alchool consumption (aOR: 4.74; aCI 95% 2.17-9.89; p < 0.001); gunshot injuries (aOR: 6.70; aCI 95% 1.84-20.11; p = 0.003), open fractures (aOR: 4.35; aCI 95% 2.17-9.12; p < 0.001), fracture site infections (aOR: 3.10; aCI 95% 1.03-7.95; p = 0.023); traditional healer (aOR: 12.18; aCI 95%: 5.74-25.37; p < 0.001), general practitioner (aOR: 8.33; aCI 95% 1.77-30.31; p = 0.006) initial absence X-ray assessment (aOR: 12.21; aCI 95% 5.92-24.96; p < 0.001). Long bone pseudarthroses are common in Butembo. The most effective way to prevent them is to know and avoid risk factors.

## Introduction

La pseudarthrose est l´une des complications tardives de la consolidation de fracture. Elle est très redoutée et très difficile à traiter, même par le chirurgien orthopédiste. Elle est très invalidante et son évolution est imprévisible [1]. Le mieux à faire est toujours de la prévenir. La pseudarthrose complique environ 1 à 5% de toutes les fractures [2]. D´après Obert, les fractures des os longs se compliquent de pseudarthrose dans 10% des cas. En cas d´appartenance à un groupe à risque (facteurs locaux et généraux présents), le taux de pseudarthrose peut atteindre 30%. En cas d´erreur technique (fixation inadéquate, contrôle approximatif de la rotation, notamment dans les fractures diaphysaires des os longs), le risque de pseudarthrose est estimé à 50% [3]. A Lausanne, en 2012, une étude sur les fractures diaphysaires du fémur menée par Maeder avait montré que le taux de pseudarthrose oscille entre 5% et 10% [4]. Dans la série de Sossou, Hans Moevi, Fiogbe, Maizar et Padonou à Cotonou en 2014, 2.94% des patients avec fracture du col fémoral avaient présenté une pseudarthrose [5]. Saad Andaloussi, Amine, Alaoui, Atarraf, Chater et Afifi ont trouvé, dans leur série de 2017 au CHU Hassan II, Fès, Maroc une fréquence de 3,4% des pseudarthroses [6].

Plusieurs facteurs sont incriminés dans la survenue de la pseudarthrose. Au niveau du patient lui-même, le revenu économique bas, l´intoxication au tabac et l´existence d´une comorbidité sont associés à la pseudarthrose [5, 7, 8]. La nature de la fracture aussi influence la survenue de la pseudarthrose, surtout lorsqu´il y a comminution ou fracture ouverte [9, 10]. Au niveau de la prise en charge, la tradithérapie est incriminée, de même qu´une ostéosynthèse imparfaite et une infection au foyer de fracture [2, 5, 8, 9, 11, 12]. Dans la pratique médicale courante à Butembo, certaines fractures se compliquent de pseudarthroses. Généralement, elles sont d´évolution difficile et à pronostic non prévisible. Il vaudrait bien la peine d´en connaitre les facteurs de risque pour les éviter, mais la littérature médicale locale est muette à ce sujet. Pour combler ce déficit, nous avons conçu de faire une étude sur les pseudarthroses à Butembo. L´objectif de cette étude était de déterminer la prévalence et les facteurs de risque des pseudarthroses traumatiques des os longs des membres en ville de Butembo, en vue d´en fournir une base de données.

## Méthodes

### Type et cadre de l´étude

Il s´agissait d´une étude rétrospective à visée descriptive et analytique sur la prévalence et les facteurs de risque des pseudarthroses des os longs des membres à Butembo. Nous avons choisi comme cadre d´étude l´Hôpital Matanda en ville de Butembo, zone de santé urbano-rurale de Katwa, Division Provinciale de Santé Nord-Kivu, à l´Est de la République Démocratique du Congo. Cet hôpital a l´avantage d´être situé en plein centre-ville de Butembo, sur la route nationale n°2. Par sa situation géographique, il est la formation sanitaire la plus accessible et la plus fréquentée de la ville, aussi bien par les habitants de la ville que par ceux des villages voisins. Il organise un département de chirurgie générale avec une section de Traumatologie et orthopédie. Un chirurgien orthopédiste y travaille à temps partiel assisté par deux infirmiers orthopédistes permanents.

### Population de l'étude

Notre population cible était constituée des patients ayant une fracture traumatique d´un os long de membre à l´hôpital Matanda, au cours de la période du 01/06/2016 au 31/05/2019, soit 968 patients. De cette population, nous avons tiré un échantillon fait de tous les patients ayant développé une pseudarthrose. Les patients sans pseudarthrose ont été considérés comme témoins. Nous avons inclus, dans l´étude, tout cas de pseudarthrose traumatique d´un os long de membre, diagnostiqué cliniquement et confirmé par une radiographie ou encore diagnostiqué en peropératoire au cours d´une ostéosynthèse ou de l´ablation d´un matériel d´ostéosynthèse. Nous avons exclu tous les patients avec pseudarthrose dont le dossier d´hospitalisation n´avait pas fourni toutes les données relatives à nos variables d´étude. Nous avons calculé la taille minimale requise pour notre échantillon à partir d´une pré-enquête à l´hôpital Matanda qui a révélé 47 cas de pseudarthroses sur 3096 cas de traumatismes des os longs des membres durant les 5 ans précédant notre étude (de 2011 à 2015), soit une prévalence de 1,5%.


$$n=\frac{Z^{2}\cdot p\left(1-p\right)}{e^{2}}$$

n= [((3,841)^2^*0,015(1-0,015))/(0,05)]^2^ =22,703856, soit 23 cas au minimum (avec: n = taille de l´échantillon; Z = coefficient de confiance à 95% qui est égale à 1,96; e = Marge d´erreur de 0,05; p= prévalence; q = complément de prévalence qui équivaut à 1-p). Nous avons répertorié 36 cas de pseudarthrose traumatique d´un os long de membre pendant notre période d´étude. Ils ont tous été inclus dans notre étude, soit un échantillon exhaustif.

### Collection de données

Les données de cette étude ont été tirées par colligation des dossiers d´hospitalisation des patients. Nous avons utilisé les variables suivantes: le type de pseudarthrose diagnostiquée et l´âge de la fracture; les caractéristiques sociodémographiques du patient: sexe, age, provenance, profession, niveau d´étude, comorbidité et intoxication; les caractéristiques initiales de la fracture: circonstance de survenue, siège (os, côté, segment de l´os), type, trait, position des fragments et infection sur le foyer ; les caractéristiques de la prise en charge initiale: qualité du soignant, radiographie, type de prise en charge initiale.

### Quelques définitions opérationnelles

1) Les âges des patients ont été groupés de façon conventionnelle par tranches de 10 ans. 2) Les anciennetés de fractures ayant présenté une pseudarthrose ont été groupées en séries de 3 mois à partir du 4^e^ mois post-fracture, en considérant que les os longs consolident généralement dans les 3 mois qui suivent la fracture. 3) Malnutrition: maigreur, cheveux soyeux, signes d´anémie et œdèmes symétriques, bilatéraux, indolores, mous et ascendants aux membres inférieurs en dehors d´un contexte de cardio-vasculopathie, hépatopathie et néphropathie.

### Analyse statistique

Le traitement des données a été réalisé par SPSS Statistics 17.0 et Stat calc Epi Info. Nous avons calculé la fréquence et utilisé un modèle de régression logistique unifactorielle et multifactorielle pour explorer les facteurs associés à la pseudarthrose. Dans l´analyse multifactorielle, nous avons inclus toutes les variables du modèle unifactoriel ayant un p ≤ 0,2, et nous avons utilisé l´Odds Ratio le plus ajusté (adjusted odds ratio), son Intervalle de confiance à 95% et son p correspondant [13].

### Considérations éthiques

L´étude était autorisée le 01 juillet 2019 par le Comité d´éthique du Nord-Kivu/CE-NK sous le numéro de protocole: 003/TEN/CENK/2019. Nous avons observé les bonnes pratiques éthiques et gardé l´anonymat des patients dans le traitement des données et la présentation des résultats.

## Résultats

### Caractéristiques générales de la population à l'étude

Dans notre étude, les patients avec fracture des os longs des membres étaient 968, dont 36 avaient présenté une pseudarthrose, soit 3,72%. De ces 36 patients, 30 étaient de sexe féminin (83,3%), 10 de la tranche d´âges 21 - 30 ans (27,7%), 25 de provenance rurale (69,4%), 22 cultivateurs (61,1%), 13 patients illettrés (36,1%) et 18 de niveau d´étude primaire (50%). La pseudarthrose la plus fréquente était le type flottant, soit 69,4%; et la plupart des fractures était d´ancienneté de 7-9 mois, soit 36,1% (Tableau 1).

**Tableau 1:** caractéristiques générales des patients

Variable	Total (N=36)	%
**Sexe**		
Féminin	30	83,3
Masculin	6	16,7
**Tranche d´âge (années)**		
≤10	1	2,8
11 - 20	1	2,8
21 - 30	10	27,7
31 - 40	5	13,9
41 - 50	5	13,9
51 - 60	5	13,9
61 - 70	8	22,2
≥71	1	2,8
**Provenance**		
Rurale	25	69,4
Urbaine	11	30,6
**Profession**		
Cultivateur	22	61,1
Ménagère	3	8,3
Pousse-pousseur	3	8,3
Chauffeur véhicule	2	5,5
Enseignant	2	5,5
Militaire/Policier	1	2,8
Constructeur	1	2,8
Taximan moto	1	2,8
Commerçant	1	2,8
**Niveau d´étude**		
Illettré	13	36,1
Primaire	18	50
Secondaire	5	13,9
Supérieur	0	0
**Type de pseudarthrose**		
Pseudarthrose flottante	25	69,44
Pseudarthrose serrée	11	30,56
**Ancienneté de la fracture (mois)**		
4 - 6	9	25
7 - 9	13	36,11
10 - 12	4	11,11
≥ 13	10	27,78

### Facteurs de risque de pseudarthrose

**Les facteurs de risque de pseudarthrose dans l´analyse unifactorielle:** l´analyse unifactorielle a montré que les facteurs suivants étaient associés à la pseudarthrose (Tableau 2): les professions pousse-pousseur et cultivateur, la comorbidité avec hypertension artérielle associée au diabète, psychose et malnutrition, l´intoxication au tabac, à alcool + tabac et à l´alcool seul, le traumatisme par balle, l´accident de travail, le faux-pas avec chute, la fracture ouverte, la présence d´un séquestre, la comminution, l´infection au foyer de fracture, le soignant tradipraticien, l´infirmier hospitalier, le médecin généraliste, l´absence de radiographie initiale, l´usage de massage-tatouage, du bandage, du fixateur externe, de la plaque vissée, de l´enclouage centromédullaire et du plâtrage.

**Tableau 2:** facteurs associés aux pseudarthroses

Variable	Total (N=968)	Cas+ n(%) (n=36)	ORb (IC à 95%)	p	ORa (IC à 95%)	P
**Profession**						
Elève/Etudiant	184	0(0)	1	0,003	1	<0,001
Pousse-pousseur	21	3(14,2)	4,62 (1,30-16,45)	0,010	4,60 (1,04-15,21)	0,023
Cultivateur	399	22(5,5)	2,31 (1,11-4,95)	0,013	2,31 (1,17-4,68)	0,008
Autres	364	11(3,0)				
**Comorbidité**						
Pas de comorbidité	775	23(3)	1	0,013	1	0,011
HTA + Diabète	4	1(25)	8,85 (0,16-112,70)	0,024		
Psychose	4	1(25)	8,85 (0,16-112,70)	0,024		
Malnutrition	30	5(16,7)	5,85 (1,63-16,97)	<0,001	5,83 (1,87-15,62)	0,004
Autres	155	6(3,9)				
**Intoxication**						
Pas d´intoxication	647	17(2,6)	1	0,011	1	0,007
Tabac<	21	4(19,1)	6,73(1,55-22,19)	<0,001	6,70 (1,84-20,11)	0,003
Alcool + Tabac	90	11(12,2)	4,75(2,03-10,43)	<0,001	4,74 (2,17-9,89)	<0,001
Alcool seul	197	3(1,5)	0,35(0,07-1,12)	0,067		
Autres	13	1(7,7)				
**Circonstance**						
Corps contondant	43	0(0)	1	0,187	1	0,094
Traumatisme par balle	21	4(19,1)	6,73 (1,55-22,19)	<0,001	6,70 (1,84-20,11)	0,003
Accident de travail	69	8(11,6)	4,08 (1,54-9,67)	<0,001	4,07 (1,68-9,10)	0,002
Faux-pas + chute	223	4(1,8)	0,41 (0,10-1,17)	0,083		
Autres	612	20(3,3)				
**Type de fracture**						
Fracture fermée	651	12(1,8)	1	<0,001	1	<0,001
Fracture ouverte	317	24(7,6)	4,36 (2,06-9,70)	<0,001	4,35 (2,17-9,12)	<0,001
**Complexité du trait**						
Trait Simple	570	6(1,1)	1	<0,001	1	<0,001
Séquestre	21	5(23,8)	9,23 (2,47-28,48)	<0,001	9,18 (2,85-25,94)	<0,001
Comminution	86	13(15,1)	6,65 (2,95-14,32)	<0,001	6,63 (3,14-13,58)	<0,001
Autres	291	12(4,1)				
**Infection au foyer**						
Infection (-)	917	31(3,4)	1	0,018	1	0.023
Infection (+)	51	5(9,8)	3,11 (1,90-8,58)	0,018	3,10 (1,03-7,95)	0,023
**Qualité du soignant**						
Chirurgien orthopédiste	882	17(1,9)	1	<0,001	1	<0,001
Tradipraticien	60	14(23,3)	12,26 (5,39-26,81)	<0,001	12,18 (5,74-25,37)	<0,001
Infirmier hospitalier	4	1(25)	8,85 (0,16-112,7)	0,024		
Médecin généraliste	13	3(23,1)	8,38 (1,41-34,45)	<0,001	8,33 (1,77-30,31)	0,006
Infirmier orthopédiste	9	1(11,1)				
**Radiographie**						
Rx initiale (+)	895	20(2,2)	1	<0,001	1	<0,001
Rx initiale (-)	73	16(21,9)	12,28 (5,59-26,34)	<0,001	12,21(5,92-24,96)	<0,001
**Technique utilisée**						
Plâtre	214	2(0,9)	1	0,015	1	0,004
Massage-tatouage	60	14(23,3)	12,26 (5,39-26,81)	<0,001	12,18 (5,74-25,37)	<0,001
Bandage	9	2(22,2)	7,77 (1,56-38,82)	0,003	7,73 (1,07-36,24)	0,023
Fixateur externe	94	6(6,4)	1,92 (0,63-4,85)	0,151		
Plaque vissée	296	7(2,4)	0,54 (0,20-1,27)	0,140		
ECM	253	4(1,6)	0,34 (0,09-0,98)	0,037		
Autres	42	1(2,4)				

ORb=Odds Ratio brut; ORa = Odds Ratio ajusté; HTA = Hypertension artérielle; Rx = Radiographie; ECM = Enclouage centromédullaire.

**Les facteurs de risque de pseudarthrose dans le modèle multifactoriel:** le modèle multifactoriel a montré que les facteurs suivants étaient significativement associés à la pseudarthrose (Tableau 2): les professions pousse-pousseur (ORa: 4,60; IC à 95% 1,04-15,21; p=0,023) et cultivateur (ORa: 2,31; IC à 95% 1,17-4,68; p = 0,008), la malnutrition (ORa: 5,83; IC à 95% 1,87-15,62 ; p = 0,004), l´intoxication au tabac (ORa: 6,70; IC à 95% 1,84-20,11; p = 0,003) et à alcool + tabac (ORa: 4,74; IC à 95% 2,17-9,89; p = 0,001), le traumatisme par balle (ORa: 6,70; IC à 95% 1,84-20,11; p = 0,003), l´accident de travail (ORa: 4,07; IC à 95% 1,68-9,10; p = 0,002), la fracture ouverte (ORa: 4,35; IC à 95% 2,17-9,12; p = 0,001), la présence d´un séquestre (ORa: 9,18; IC à 95% 2,85-25,94; p = 0,001), la comminution (ORa: 6,63; IC à 95% 3,14-13,58; p = 0,001), l´infection au foyer de fracture (ORa: 3,10; IC à 95% 1,03-7,95; p = 0,023); le soignant tradipraticien (ORa: 12,18; IC à 95% 5,74-25,37; p = 0,001), le médecin généraliste (ORa: 8,33; IC à 95% 1,77-30,31; p=0,006), l´absence de radiographie initiale (ORa: 12,21; IC à 95% 5,92-24,96; p = 0,001), l´usage de massage-tatouage (ORa: 12,18; IC à 95% 5,74-25,37; p = 0,001) et du bandage (ORa: 7,73; IC à 95% 1,07-36,24 ; p = 0,023).

## Discussion

L´objectif de notre étude était de déterminer la prévalence et les facteurs de risque des pseudarthroses traumatiques des os longs des membres en ville de Butembo. La prévalence des pseudarthroses des os longs des membres était de 3,72% et plusieurs facteurs de risque ont été trouvés. Ce résultat correspond à la fréquence de 1 à 5% que représentent les pseudarthroses au sein de toutes les fractures selon Thein, Ookera et Everding [2, 12, 14]. La pseudarthrose flottante était le type de pseudarthrose le plus fréquent. Dans sa série, Aniss avait trouvé 73,3% de pseudarthrose lâche [11]. Pour Sossou, les écarts interfragmentaires observés et mesurés sur tous les clichés de pseudarthrose étaient de 5 mm dans 15% des cas, 6 mm dans 46% des cas et 4 mm dans 31% des cas et 1 mm dans 8% des cas [5]. Dans notre étude, les fractures anciennes de 7 à 9 mois étaient les plus fréquentes, suivies de celles anciennes de plus de 13 mois. Chez Aniss, le délai entre la fracture initiale et la consultation pour une prise en charge de pseudarthrose variait entre 3 et 18 mois [11].

Les pousse-pousseurs et les cultivateurs étaient des groupes professionnels à risque dans notre étude. Ce sont des groupes à faibles revenus financiers dans notre pays. Sossou avait trouvé que le manque de moyen financier conduisait les patients à refuser le traitement chirurgical classique [5]. La malnutrition était aussi un facteur de risque dans notre étude. Einhorn avait indiqué la comorbidité parmi les facteurs de risque de survenue d'une pseudarthrose [8]. Les auteurs spécialisés Ookera avaient mentionné l'existence de maladies chroniques comme le diabète et le statut nutritionnel comme facteurs de risque [12]. L´intoxication au tabac/drogue et à l´alcool associé au tabac/drogue était également un facteur de risque de pseudarthrose. Pour Fabre, le tabagisme était particulièrement péjoratif [9]. Pour Thein et les auteurs specialisés Ookera aussi, le tabagisme et l´alcoolisme chronique étaient aussi des facteurs de risque [2, 12]. Dans la série de Prommersbergerl, 26,7% des patients étaient des fumeurs de tabac [7]. Pour Budzik également, le tabagisme chronique était un facteur de risque connu de pseudarthrose [15].

Dans notre étude, le traumatisme par balle et les fractures ouvertes, avec séquestre ou avec comminution étaient aussi significativement associées au risque de pseudarthrose. Dans la série de Said, le caractère comminutif du foyer était un facteur de pseudarthrose [10]. Selon Fabre, les lésions osseuses avec ouverture du foyer font toujours basculer le pronostic car les lésions des parties molles (délabrements, contusions et dévascularisation) sont des facteurs de gravité pouvant conduire à une pseudarthrose [9]. Dans son étude, Mohammed Elibrissi avait incriminé une vascularisation médiocre qui peut être liée à une ouverture initiale, un déperiostage excessif ou la persistance d'un defect osseux [8].

L´infection du foyer de fracture était aussi un facteur significatif de pseudarthrose. Dans les études respectives de Mohammed Elibrissi, de Thein et des auteurs spécialisés Ookera, l´infection était retenue comme facteur de risque de pseudarthrose [2, 8, 12]. Le personnel soignant tradipraticien dans sa case au village et même le médecin généraliste, l´absence de la radiographie, le massage-tatouage et le bandage ont été des facteurs de risque dans notre étude. Aniss avait indiqué que la pseudarthrose était due soit à une négligence thérapeutique, soit à une ostéosynthèse imparfaite [11]. Pour Fabre, l´incompétence du chirurgien était l´une des causes de pseudarthrose [9]. Gonschorek avait parlé en termes de solutions techniques sous-optimales à la fracture pouvant être le facteur déclencheur d´une pseudarthrose [16]. Bouattour et Altolnan avaient respectivement montré que le traitement traditionnel était la première cause de pseudarthrose dans leurs séries des pseudarthroses sur fracture du fémur, soit 75% et 81% [5]. Dans la serie de Sossou, 16% des pseudarthroses étaient conséquence de la tradithérapie [5]. Prommersberger avait trouvé 6,7% des patients avec pseudarthrose traités initialement de manière non opératoire [7]. Par contre, l´Enclouage centromédullaire (ECM) et la plaque-vissée sont des techniques qui ont porté des bons résultats aussi bien dans notre étude que chez plusieurs auteurs d´après la littérature [10, 17-19].

Notre étude a eu le privilège de rendre disponibles les données locales sur la pseudarthrose et surtout d´en identifier les facteurs de risque évitables. Grâce au modèle multifactoriel utilisé, nos conclusions sont généralisables à toute la population de Butembo. Cependant, étant rétrospective, cette étude s´est limitée aux données contenues dans les dossiers des patients dont certains ont été exclus de l´étude, car incomplets ou mal remplis. Il aurait été mieux encore de faire une étude longitudinale et prospective des pseudarthroses à partir des fractures fraiches. Par ailleurs, notre étude n´a pas intégré les pseudarthroses d´origine infectieuse, notamment post ostéomyélite.

## Conclusion

A Butembo, certaines fractures se compliquent de pseudarthrose et le type flottant est le plus représenté. Les personnes les plus exposées à la pseudarthrose sont les pousse-pousseurs, les cultivateurs, les malnutris, les tabagiques et les alcooliques. Les lésions par armes à feu, la fracture ouverte avec séquestre ou avec comminution, la fréquentation des tradipratitiens, le manque de radiographie et l´infection du foyer, concourent significativement à la survenue des pseudarthroses. Nous pensons que la connaissance et l´éviction de ces facteurs de risque de pseudarthrose pourraient en être une voie de solution efficace.

### Etat des connaissances sur le sujet

- La pseudarthrose représente environ 1 à 5% de toutes les fractures et 10% des fractures des os longs. En cas d´appartenance à un groupe à risque (facteurs locaux et généraux présents), le taux de pseudarthrose peut atteindre 30%;
- La pseudarthrose est très difficile à traiter;
- Les facteurs de risque les plus fréquemment rencontrés sont: le revenu économique bas, l´intoxication au tabac, la comorbidité, la fracture comminutive, la fracture ouverte, la tradithérapie, l´ostéosynthèse imparfaite et l´infection.

### Contribution de notre étude à la connaissance

- La prévalence des pseudarthroses traumatiques des os des membres est de 3,72% à Butembo;
- Les facteurs associés à la pseudarthrose traumatique des os longs des membres à butembo sont: les professions pousse-pousseur et cultivateur, la malnutrition, l´intoxication au tabac et à alcool, le traumatisme par balle, la fracture ouverte, la présence d´un séquestre et la comminution, l´infection au foyer de fracture, le soignant tradipraticien, le médecin généraliste et l´absence de radiographie initiale.

## Remerciements

Nous remercions toutes les personnes qui nous ont soutenus et encouragés dans la réalisation et la publication de cette étude.
